# Hearing loss in Australian First Nations children at 6-monthly assessments from age 12 to 36 months: Secondary outcomes from randomised controlled trials of novel pneumococcal conjugate vaccine schedules

**DOI:** 10.1371/journal.pmed.1004375

**Published:** 2024-06-03

**Authors:** Amanda Jane Leach, Nicole Wilson, Beth Arrowsmith, Jemima Beissbarth, Kim Mulholland, Mathuram Santosham, Paul John Torzillo, Peter McIntyre, Heidi Smith-Vaughan, Sue A. Skull, Victor M. Oguoma, Mark D. Chatfield, Deborah Lehmann, Christopher G. Brennan-Jones, Michael J. Binks, Paul V. Licciardi, Ross M. Andrews, Tom Snelling, Vicki Krause, Jonathan Carapetis, Anne B. Chang, Peter Stanley Morris

**Affiliations:** 1 Menzies School of Health Research, Charles Darwin University, Darwin, Northern Territory, Australia; 2 Telethon Kids Institute, University of Western Australia, Perth, Western Australia, Australia; 3 London School of Hygiene and Tropical Medicine, London, United Kingdom; 4 Department of Paediatrics, University of Melbourne, Melbourne, Victoria, Australia; 5 Murdoch Children’s Research Institute, Melbourne, Victoria, Australia; 6 Departments of International Health and Pediatrics, Johns Hopkins Bloomberg School of Public Health, Baltimore, Maryland, United States of America; 7 Center for Indigenous Health, Johns Hopkins University, Baltimore, Maryland, United States of America; 8 Royal Prince Alfred Hospital, Camperdown, New South Wales, Australia; 9 Department of Medicine, University of Sydney, Sydney, New South Wales, Australia; 10 Discipline of Child and Adolescent Health, University of Sydney, Sydney, New South Wales, Australia; 11 Department of Women’s and Children’s Health, Dunedin School of Medicine, University of Otago, Dunedin, New Zealand; 12 Division of Paediatrics and Child Health, University of Western Australia, Perth, Western Australia, Australia; 13 Poche Centre for Indigenous Health, The University of Queensland, Brisbane, Queensland, Australia; 14 Faculty of Medicine, University of Queensland, Brisbane, Queensland, Australia; 15 School of Allied Health, Faculty of Health Sciences, Curtin University, Perth, Western Australia, Australia; 16 Women and Kids Theme, South Australian Health and Medical Research Institute, Adelaide, South Australia, Australia; 17 Office of the Chief Health Officer, Queensland Health, Brisbane, Queensland, Australia; 18 School of Public Health, University of Sydney, Sydney, New South Wales, Australia; 19 Centre for Disease Control (CDC)-Environmental Health, Northern Territory Health, Darwin, Northern Territory, Australia; 20 Australian Centre for Health Services Innovation, Queensland University of Technology, Brisbane, Queensland, Australia; 21 Paediatrics Department, Royal Darwin Hospital, Darwin, Northern Territory, Australia

## Abstract

**Background:**

In Australian remote communities, First Nations children with otitis media (OM)-related hearing loss are disproportionately at risk of developmental delay and poor school performance, compared to those with normal hearing. Our objective was to compare OM-related hearing loss in children randomised to one of 2 pneumococcal conjugate vaccine (PCV) formulations.

**Methods and findings:**

In 2 sequential parallel, open-label, randomised controlled trials (the PREVIX trials), eligible infants were first allocated 1:1:1 at age 28 to 38 days to standard or mixed PCV schedules, then at age 12 months to PCV13 (13-valent pneumococcal conjugate vaccine, +P) or PHiD-CV10 (10-valent pneumococcal *Haemophilus influenzae* protein D conjugate vaccine, +S) (1:1). Here, we report prevalence and level of hearing loss outcomes in the +P and +S groups at 6-monthly scheduled assessments from age 12 to 36 months. From March 2013 to September 2018, 261 infants were enrolled and 461 hearing assessments were performed. Prevalence of hearing loss was 78% (25/32) in the +P group and 71% (20/28) in the +S group at baseline, declining to 52% (28/54) in the +P groups and 56% (33/59) in the +S group at age 36 months. At primary endpoint age 18 months, prevalence of moderate (disabling) hearing loss was 21% (9/42) in the +P group and 41% (20/49) in the +S group (difference −19%; (95% confidence interval (CI) [−38, −1], *p* = 0.07) and prevalence of no hearing loss was 36% (15/42) in the +P group and 16% (8/49) in the +S group (difference 19%; (95% CI [2, 37], *p* = 0.05). At subsequent time points, prevalence of moderate hearing loss remained lower in the +P group: differences −3%; (95% CI [−23, 18], *p* = 1.00 at age 24 months), −12%; (95% CI [−30, 6], *p* = 0.29 at age 30 months), and −9%; (95% CI [−23, 5], *p* = 0.25 at age 36 months). A major limitation was the small sample size, hence low power to reach statistical significance, thereby reducing confidence in the effect size.

**Conclusions:**

In this study, we observed a high prevalence and persistence of moderate (disabling) hearing loss throughout early childhood. We found a lower prevalence of moderate hearing loss and correspondingly higher prevalence of no hearing loss in the +P group, which may have substantial benefits for high-risk children, their families, and society, but warrant further investigation.

**Trial registration:**

ClinicalTrials.gov NCT01735084 and NCT01174849

## Abstract 2

Amanda Leach and colleagues compare two pneumococcal conjugate vaccines to examine the impact on hearing loss

## Author summary

**Why was this study done?:**

- Chronic otitis media (OM) (middle ear infection) causes hearing loss, which is linked to developmental delay, poor school readiness, attendance, and performance.
- Almost every Australian First Nations child living in remote regions experiences chronic OM throughout their formative early years.
- Vaccines effective against OM pathogens, namely, pneumococcus and nontypeable *Haemophilus influenzae*, could reduce chronic early childhood hearing loss and change the trajectory of disadvantage.
- Two pneumococcal conjugate vaccines with different formulations potentially protect from infection by either 13 pneumococcal strains (PCV13), or 10 of these 13 pneumococcal strains plus all nontypeable *H*. *influenzae* (PHiD-CV10).
- Our hypothesis was that PHiD-CV10 would be superior to PCV13 in preventing hearing loss.
- No study had compared PCV13 and PHiD-CV10 in head-to-head or combination schedules.

**What did the researchers do and find?:**

- We designed 2 consecutive (primary course then booster dose) randomised controlled trials of head-to-head and combination PCV13 and PHiD-CV10 schedules. Audiologists assessed hearing at 6-monthly visits from age 12 to 36 months.
- Moderate (disabling) hearing loss was detected in 42% (25/60), 32% (29/91), 34% (29/86), 33% (37/111), and 19% (22/113) children at ages 12, 18, 24, 30, and 36 months, respectively.
- Hearing Australia’s recommendation for hearing assistance was met in around 70% children at ages 12 (67%, 40/60), 18 (69%, 63/91), and 24 months (71%, 61/86), respectively.
- Contrary to our hypothesis, PHiD-CV10 vaccine was not superior to PCV13 in reducing hearing loss.
- At primary endpoint age 18 months, the prevalence of moderate (disabling) hearing loss was halved in the PCV13 group (21% (9/42) compared to 41% (20/49) in the PHiD-CV10 group, and the prevalence of no hearing loss was double (36% and 16%, respectively).
- At ages 24, 30, and 36 months, this trend continued but differences were smaller.

**What do these findings mean?:**

- The 19% lower prevalence of moderate hearing loss and 19% higher prevalence of no hearing loss in the PCV13 group at age 18 months is potentially highly clinically significant, particularly as the trend appears to be sustained, albeit smaller.
- Our study emphasises the importance of innovative and targeted research designs that meet the needs of high-risk populations and are independent of the pharmaceutical industry.
- Pneumococcal conjugate vaccine trials should evaluate impacts on child development.
- The small sample size creates uncertainty about the plausible difference in hearing between the two vaccines. We are 95% confident that the true difference is between 1% and 37%.

## Introduction

### Background and objectives

Australian First Nations children living in remote communities continue to experience social and educational disadvantage [1], which can be attributed in part to preventable hearing loss associated with early onset of persistent otitis media (OM). *Streptococcus pneumoniae* (pneumococcus) and nontypeable *Haemophilus influenzae* (NTHi) are dominant pathogens of OM from soon after birth [2].

The Northern Territory (NT) childhood vaccination schedule replaced 7-valent pneumococcal conjugate vaccine (PCV7) with 10-valent pneumococcal *H*. *influenzae* protein D conjugate vaccine (S, PHiD-CV10) in 2009, then 13-valent PCV (P, PCV13) vaccine in 2011. Our surveillance studies found evidence of less acute otitis media (AOM) and a lower prevalence of NTHi in ear discharge of PHiD-CV10-vaccinated compared to PCV7-vaccinated First Nations children [2,3].

Our objective was to conduct 2 sequential randomised controlled trials (RCTs) of primary and booster head-to-head and combination schedules of PCV13 and PHiD-CV10; PREVIX_COMBO [4,5] and PREVIX_BOOST [6], PREVIX being a name representing the 2 PCV brand names.

We hypothesised that infants receiving PHiD-CV10 compared with those receiving PCV13 as a booster at 12 months of age would have superior outcomes in terms of immunogenicity, nasopharyngeal carriage, OM, respiratory illness, hearing loss, and developmental delay [7]. We have reported coprimary and select secondary outcomes to age 18 months [4,6,8] and OM to age 36 months [9].

This study, PREVIX_VOICES (Vaccines for Otitis In Children Entering School), builds on PREVIX_BOOST through added capacity to employ paediatric audiologists to conduct 6-monthly hearing tests from age 12 to 36 months. Here, we report new data on vaccine group comparisons of hearing level and prevalence of hearing loss.

## Methods

This study is reported as per Consolidated Standards of Reporting Trials (CONSORT) guideline (S1 CONSORT Checklist) [10]. Details of PREVIX_COMBO and PREVIX_BOOST (including PREVIX_VOICES) protocols have been published [5,7]. Brief methods relevant to hearing outcomes from age 12 to 36 months are described below.

### Study design

The PREVIX_COMBO and PREVIX_BOOST trials were primary outcome assessor-blinded, 3-arm (1:1:1) and 2-arm (1:1) parallel RCTs. PREVIX_VOICES added capacity to obtain the hearing outcomes for all children in the PREVIX_BOOST study and additional scheduled 6-monthly assessments at ages 24 to 30 months. No changes that affected the primary objectives or outcomes were made after trial commencement.

### Participants

First Nations children were eligible for PREVIX_BOOST if they were 12 months of age, had previously enrolled in the PREVIX_COMBO 3-arm RCT, and were living in one of 3 remote Aboriginal communities in the NT or a single Western Australian community. PREVIX_VOICES evaluated hearing outcomes in all PREVIX-enrolled children living in the 3 NT communities.

### Randomisation and masking

Sequence generation: After confirming eligibility and obtaining informed consent, research nurses called the 24/7 randomisation service of the National Health and Medical Research Council Clinical Trial Centre to obtain allocation. Stratification by remote community was applied in both trials, and for PREVIX_BOOST, minimisation by PREVIX_COMBO group was applied. This open-label design did not allow for blinding.

### Procedures

PHiD-CV10 contains 10 pneumococcal serotypes 1, 4, 5, 6B, 7F, 9V, 14, 18C, 19F, 23F and incorporates protein D of NTHi as a carrier protein for 8 serotypes. PCV13 contains these 10 and additional serotypes 3, 6A, and 19A but does not incorporate protein D.

Audiology assessments were initially made by fly-in/fly-out NT Government Hearing Health Program audiologists. Paediatric audiologists dedicated to assessment of hearing in the PREVIX_VOICES study commenced in March 2017. All assessments were conducted in the child’s community, in sound-treated hearing booths using an audiometer (Interacoustics paediatric audiometer (PA5) or Otometrics MADSEN Itera II). Hearing responses were tested using Visual Reinforcement Observation Audiometry (VROA) in the sound-field or Play Audiometry using headphones. The pure-tone 4-frequency average (4fa) hearing level (in decibels, dB HL) was calculated for air conduction thresholds binaural or monaurally using up to 4 frequencies 0·5, 1·0, 2·0, and 4·0 kHz (i.e., ≤4fa). Categories of hearing response (hereafter referred to as hearing loss) were no or slight hearing loss (≤0 to 20 dB), mild (21 to 30 dB), moderate (31 to 60 dB), severe (61 to 90 dB), or profound (>90 dB) hearing loss [11]. We also report the proportion with mean hearing level (3fa) greater than 23.3 dB, which is the Hearing Australia threshold for hearing assistance.[12] All child-visits included a general health check, with management according to local guidelines [13,14].

### Analysis and statistical methods

Sample sizes of 425 and 270 were estimated for primary outcomes (immunogenicity) of PREVIX_COMBO and PREVIX_BOOST, respectively [4,6]. Analyses of secondary outcome data available at each time point are according to allocated group. We did not impute any data. All data were analysed using Stata/IC version 15.1 [15]. Baseline characteristics are reported for each PREVIX_BOOST group using means and standard deviation for continuous data if assumption of normal distribution was met; otherwise, median value and IQR are reported. Categorical data are summarised as percentages. Vaccine group differences in percentages are compared with 2-sided Clopper–Pearson 95% confidence interval (95% CI, Fisher’s exact *p*-value) and differences in mean hearing level as 2-sample *t* test with equal variances.

## Results

The PREVIX_BOOST trial commenced participant recruitment in March 2013. Hearing tests were performed by the NT Government Hearing Services outreach program if possible. Research audiologists commenced in March 2017. Data collection was completed in September 2018.

At baseline age 12 months, 131 children were allocated to +P and 130 to +S; 1 child allocated to +P received +S and was included in the +P group for analysis. Of 461 hearing tests, 409 (89%) were VROA and 52 (11%) Play Audiometry; 74% were performed by research audiologists. Very few children were lost to follow-up for outcome measures other than hearing assessments.

There were no significant vaccine intergroup differences in characteristics of children in the hearing cohort at any age, other than at age 24 months when the +S group had a higher baseline proportion of siblings with “runny ears” (32%) than the +P group (13%), and at age 36 months when the +S group had a higher baseline proportion of breast feeding in infancy (73%) than the +P group (52%). (Table 1)

**Table 1:** Baseline characteristics and risk factors for the hearing cohort at each study visit age.

Vaccine group		+P	+S	+P	+S	+P	+S	+P	+S	+P	+S
**Age at hearing test, months (SD)**		**12·7 (1·1)**	**12·6 (1·4)**	**18·4 (1·1)**	**18·5 (1·4)**	**24·1 (1·2)**	**24·3 (1·2)**	**30·2 (1·5)**	**30·3 (1·4)**	**36·9 (1·9)**	**36·9 (1·8)**
**N**		**32**	**28**	**42**	**49**	**47**	**39**	**55**	**56**	**54**	**59**
Sex	Male	16/32 (50%)	14/28 (50%)	19/42 (45%)	24/49 (49%)	22/47 (47%)	19/39 (49%)	29/55 (53%)	36/56 (64%)	27/54 (50%)	37/59 (63%)
Gestational age at birth, weeks (SD)		38·2 (1·2)	38·1 (2·0)	38·3 (1·2)	38·0 (1·7)	37·9 (1·5)	38·2 (1·7)	38·1 (1·2)	38·1 (1·5)	38·0 (1·5)	38·2 (1·5)
Birth weight, kg (SD)		3·08 (0·48)	3·17 (0·49)	3·06 (0·49)	3·17 (0·49)	3·13 (0·56)	3·13 (0·44)	3·02 (0·48)	3·11 (0·46)	3·14 (0·52)	3·12 (0·38)
Any OM (otoscopy and tympanometry)	Yes	25/30 (83%)	25/26 (96%)	38/41 (93%)	43/48 (90%)	31/42 (74%)	31/35 (89%)	39/53 (76%)	40/50 (80%)	30/51 (59%)	39/56 (70%)
Were you breastfeeding?	Yes	31/32 (97%)	25/27 (93%)	30/35 (86%)	34/41 (83%)	38/46 (83%)	30/39 (77%)	37/55 (67%)	43/55 (78%)	28/54 (52%)	43/59 (73%)
Community	Wurrumiyanga	11/32 (34%)	8/28 (29%)	10/42 (24%)	14/49 (29%)	15/47 (32%)	12/39 (31%)	13/55 (24%)	16/56 (29%)	11/54 (20%)	16/59 (27%)
	Wadeye	14/32 (44%)	13/28 (46%)	22/42 (52%)	20/49 (41%)	20/47 (43%)	14/39 (36%)	21/55 (38%)	23/56 (41%)	26/54 (48%)	26/59 (44%)
	Maningrida	7/32 (22%)	7/28 (25%)	10/42 (24%)	15/49 (31%)	12/47 (26%)	13/39 (33%)	21/55 (38%)	17/56 (30%)	17/54 (31%)	17/59 (29%)
How many children under age 5 years live with you and your baby?	0	14/32 (44%)	11/28 (39%)	18/42 (43%)	20/49 (41%)	25/47 (53%)	20/39 (51%)	28/55 (51%)	23/56 (41%)	20/54 (37%)	28/59 (47%)
	1	10/32 (31%)	5/28 (18%)	15/42 (36%)	13/49 (27%)	12/47 (26%)	12/39 (31%)	14/55 (25%)	18/56 (32%)	17/54 (31%)	18/59 (31%)
	>1	8/32 (25%)	12/28 (43%)	9/42 (21%)	16/49 (33%)	10/47 (21%)	7/39 (18%)	13/55 (24%)	15/56 (27%)	17/54 (31%)	13/59 (22%)
Have any of your other children had runny ears?	Yes	3/21 (14%)	3/23 (13%)	7/33 (21%)	10/36 (28%)	5/40 (13%)	10/31 (32%)	9/45 (20%)	13/43 (30%)	12/49 (24%)	8/48 (17%)
Has your baby had any bad runny nose since the last study visit?	Yes	12/32 (38%)	12/28 (43%)	19/42 (45%)	29/48 (60%)	19/47 (40%)	17/39 (44%)	27/55 (49%)	21/56 (38%)	23/53 (43%)	35/59 (59%)
Do you smoke cigarettes?	Yes	20/32 (63%)	17/27 (63%)	27/41 (66%)	27/48 (56%)	28/46 (61%)	23/39 (59%)	36/55 (65%)	35/54 (65%)	37/54 (69%)	36/59 (61%)

**Fig 1:**
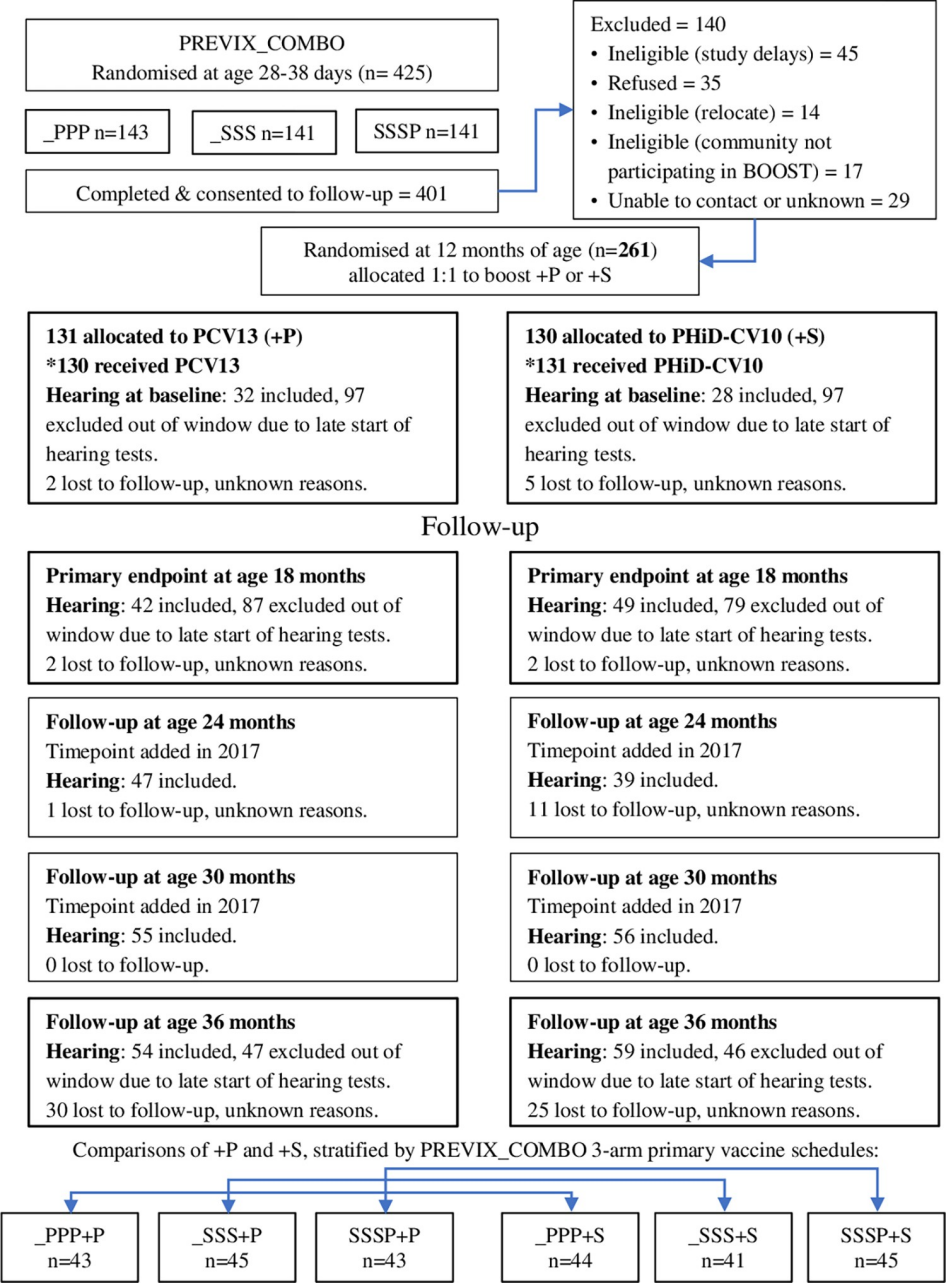
Participant flow. P: PCV13 13-valent pneumococcal conjugate vaccine. S: PHiD-CV10 10-valent pneumococcal *H*. *influenzae* protein D conjugate vaccine. *One child allocated to +P received +S.

We report the numbers of children assessed by audiologists as a proportion of all children followed up within window by research nurses for any study outcome measure; only 23% PREVIX_BOOST children were age-eligible for hearing assessments at age 12 months, 35% at age 18 months, and around half (55%) at age 36 months. From 2017, for the additional study visits introduced at ages 24 and 30 months, 88% age-eligible children had hearing assessments at age 24 months, and 100% at 30 months (Table 2 and Fig 1).

**Table 2:** Proportion of children with hearing loss by category (normal, mild, moderate, severe, >23·3 dB), and mean hearing loss (dB, SD) at ages 12 (baseline), 18, 24, 30, and 36 months, by booster dose (difference, 95% CI, *p*-value).

Visit age (months)	Hearing loss category or level (4fa dB, SD)	Total	+P	+S	DIFF +P—+S	95% CI	*P* value
**12**	Tested/allocated	60/261 (23%)	32/131 (24%)	28/130 (22%)	3%	−7, 13	0·66
	None	15 (25%)	7 (22%)	8 (29%)	−7%	−29, 15	0·57
	Mild	20 (33%)	11 (34%)	9 (32%)	2%	−22, 26	1·00
	Moderate	25 (42%)	14 (44%)	11 (39%)	4%	−20, 29	0·80
	>23·3 (3fa)	40 (67%)	22 (69%)	18 (64%)	4%	−19, 28	0.79
	Mean dB (SD)	29·5 (8·4)	29·5 (8·2)	29·5 (8·8)	0·02 dB	−4·4, 4·4	1·00
**18**	Tested/followed-up	91/257(35%)	42/129 (33%)	49/128 (38%)	−6%	−17, 6	0·36
	None	23 (25%)	15 (36%)	8 (16%)	19%	2, 37	0·05
	Mild	39 (43%)	18 (43%)	21 (43%)	0%	−20, 20	1·00
	Moderate	29 (32%)	9 (21%)	20 (41%)	−19%	−38, -1	0·07
	>23·3 (3fa)	63 (69%)	26 (62%)	37 (76%)	−14%	−33, 5	0.18
	Mean dB (SD)	28·0 (7·9)	26·2 (6·9)	29·6 (8·4)	**−3·4 dB**	−6·7, −0·2	**0·04**
**24**	Tested/followed-up	86/98 (88%)	47/48 (98%)	39/50 (78%)	20%	8, 32	0·004
	None	19 (22%)	11 (23%)	8 (21%)	3%	−15, 20	0·80
	Mild	38 (44%)	21 (45%)	17 (44%)	−1%	−20, 22	1·00
	Moderate	29 (34%)	15 (32%)	14 (36%)	−3%	−23, 18	1·00
	>23·3 (3fa)	61 (71%)	31 (66%)	30 (77%)	−11%	−30, 8	0.34
	Mean dB (SD)	28·9 (9·4)	27·7 (8·1)	30·3 (10·7)	−2·6 dB	−6·6, 1·4	0·20
**30**	Tested/followed-up	111/111 (100%)	55/55 (100%)	56/56 (100%)	0%	na	na
	None	42 (38%)	24 (44%)	18 (32%)	12%	−6, 29	0·24
	Mild	32 (29%)	16 (29%)	16 (29%)	0%	−16, 17	1·00
	Moderate	37 (33%)	15 (27%)	22 (39%)	−12%	−30, 6	0·29
	>23·3 (3fa)	56 (50%)	26 (47%)	30 (54%)	−6%	−25, 12	0.57
	Mean dB (SD)	26·9 (9·4)	26.0 (8·7)	27·8 (10·1)	−1·9 dB	−5·4, 1·7	0·30
**36**	Tested/followed-up	113/206 (55%)	54/101 (53%)	59/105 (56%)	3%	−11, 17	0.67
	None	51 (45%)	26 (48%)	25 (42%)	6%	−13, 12	0·57
	Mild	39 (34%)	20(37%)	19 (32%)	5%	−13, 22	0·69
	Moderate	22 (19%)	8 (15%)	14 (24%)	−9%	−23, 5	0·25
	Severe	1 (0·6%)	0 (0%)	1 (1·7%)	−1%	−3, 1	1·00
	>23·3 (3fa)	49 (43%)	22 (41%)	27 (45%)	−4%	−22, 14	0.71
	Mean dB (SD)	24·5 (10·4)	22·8 (7·2)	25·9 (12·5)	−3·1 dB	−7.0, 0·7	0·11

**Bold** indicates significant vaccine group difference *p* < 0·05.

+P: booster dose of PCV13 13-valent pneumococcal conjugate vaccine.

+S: booster dose of PHiD-CV10 10-valent pneumococcal *H*. *influenzae* protein D conjugate vaccine.

CI, confidence interval; dB, decibel; SD, standard deviation.

Hearing loss categories: None ≤20 dB. Mild 21 to 30 dB. Moderate 31 to 60 dB. Severe 61 to 90 dB. Profound >90 dB.

4fa: average of up to 4 frequencies (500, 1,000, 2,000, and 4,000 Hertz).

>23·3 (3fa): Hearing Australia threshold for hearing assistance.

Tested: the numbers of participants who had a hearing test.

Followed-up: the number of participants who had a hearing test plus those excluded.

*P* value. 2-sided Fisher’s exact, or *t* test.

**Fig 2:**
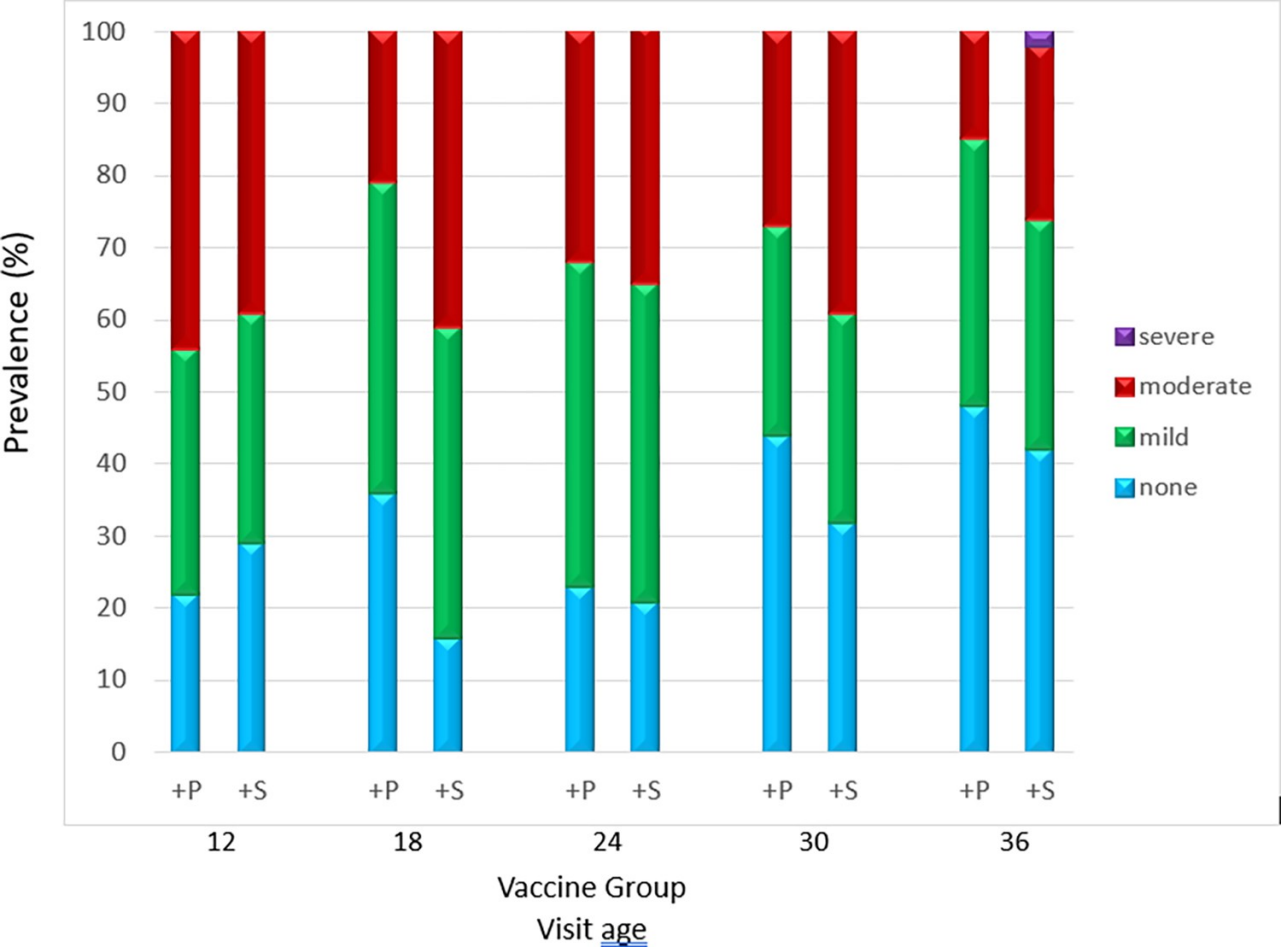
Hearing loss prevalence (none, mild, moderate, severe) by booster dose, at ages 12 (baseline), 18 (primary endpoint), 24, 30, and 36 months. +P: booster dose of PCV13 13-valent pneumococcal conjugate vaccine. +S: booster dose of PHiD-CV10 10-valent pneumococcal *H*. *influenzae* protein D conjugate vaccine.

At baseline age 12 months, 32/131 (24%) children in the +P and 28/130 (22%) in the +S group had hearing tests; hearing loss was similar in both groups. (Table 2 and Fig 2) At age 18 months, 6 months post booster dose, 42 children in the +P group and 49 in the +S group had hearing tests; 36% (+P) and 16% (+S) had no hearing loss (difference 19%; (95% CI [2, 37], *p* = 0·05); 43% in each group had mild hearing loss, and 21% (+P) and 41% (+S) had moderate hearing loss (difference −19%; (95% CI [−38, −1], *p* = 0·07) (Table 2 and Fig 2). Mean hearing level was 26·2 dB (+P) and 29·6 dB (+S), (difference −3·4 dB; (95% CI [−6·7, −0·2] *p* = 0·04) (Table 2). The Hearing Australia threshold for recommended hearing assistance (mean hearing level (3fa) greater than 23·3 dB) [12] was met by 62% (+P) and 76% (+S) children (difference −14%; (95% CI [−33, 5] *p* = 0·18) (Table 2).

At ages 24, 30, and 36 months, the proportions of children with no hearing loss was higher, mean hearing level and moderate hearing loss tended to be lower in the +P group (Table 2 and Fig 2).

Analysis stratified by allocation to 3 primary course schedules in PREVIX_COMBO indicated that the lower prevalence of moderate hearing loss in the +P group was present in all subgroups (differences −8% between _PPP+P and _PPP+S, −5% between _SSS+P and _SSS+S, and −15% between SSSP+P and SSSP+S). The greatest difference was between SSSP+P (23%) and SSSP+S (38%) (difference −15%; (95% CI [−30, −0.1], *p* = 0·07) (Table 3).

**Table 3:** Prevalence of hearing loss in the +P versus +S groups, stratified by allocation to 3 primary course PCV schedules (_PPP, _SSS, and SSSP).

Primary course	_PPP			_SSS			SSSP			TOTAL
**Booster dose**	**+P**	**+S**		**+P**	**+S**		**+P**	**+S**		
**N**	80	63		85	91		65	76		460
**Category of hearing loss**	n (%)	n (%)	Diff [95% CI] *p*-value	n (%)	n (%)	Diff [95% CI] *p*-value	n (%)	n (%)	Diff [95% CI] *p*-value	
**None (<20 dB)**	26 (33%)	17 (27%)	6% [−10, 21] 0.58	31 (36%)	25 (27%)	9% [−5, 23] 0.26	26 (40%)	25 (33%)	7% [−9, 23] 0.48	150 (33%)
**Mild (21–30 dB)**	31 (39%)	23 (37%)	2% [−14, 18] 0.86	31 (36%)	37 (41%)	−4% [−19, 10] 0.64	24 (37%)	22 (29%)	8% [−7, 24] 0.37	168 (37%)
**Moderate (31–60 dB)**	23 (29%)	23 (37%)	−8% [−23, 8] 0.37	23 (27%)	29 (32%)	−5% [−18, 9] 0.51	15 (23%)	29 (38%)	−15% [−30, −0.1] 0.07	142 (31%)

Fluctuating hearing loss was examined using data from children who had hearing tests at 2 or more consecutive 6-monthly assessments. Overall, at initial assessment, the proportion of hearing tests that found no hearing loss (35%), mild (36%), or moderate (29%) hearing loss was similar to 6-monthly follow-up (38%, 36%, 25%, respectively). However, transitions in hearing status were more dynamic than revealed by point prevalence: Of 73 children with no hearing loss initially, 51% had no hearing loss at follow-up, 37% transitioned to mild, and 12% to moderate hearing loss. Of 76 children with mild hearing loss initially, 31% transitioned to no hearing loss, 42% had persisting mild hearing loss, 26% transitioned to moderate hearing loss, and 1% to severe hearing loss. Of 61 children with moderate hearing loss initially, 33% transitioned to no hearing loss, 28% transitioned to mild hearing loss, and 39% had persisting moderate hearing loss (Table 4).

**Table 4:** Transitions between consecutive 6-monthly hearing assessments.

Hearing loss category after 6 months:	Normal: <20 dB	Mild: 21 to 30 dB	Moderate: 31 to 60 dB	Severe: 61 to 90 dB	Total
**Hearing loss category at initial assessment:**					
**Normal: <20 dB**	37 51%	27 37%	9 12%	0	73 (35%)
**Mild: 21 to 30 dB**	24 31%	32 42%	20 26%	1 (1%)	77 (36%)
**Moderate: 31 to 60 dB**	20 33%	17 28%	24 39%	0	61 (29%)
**Total**	81 (38%)	76 (36%)	53 (25%)	1 (0.5%)	211 (100%)

## Discussion

To our knowledge, this is the first study globally to evaluate hearing loss outcomes in an RCT of head-to-head or mixed schedule PCVs. This is also the first prospective longitudinal community-based study of scheduled hearing tests in First Nations children from age 12 to 36 months. A literature search for studies of either PCV13 or PHiD-CV10 that report either OM or hearing-related outcomes [14] was repeated for the period 2019 to 2023; of 54 identified studies, none reported hearing outcomes. A recent 2020 Cochrane review of PCVs for prevention of acute OM did not report hearing outcomes [16]. Other head-to-head RCTs compare immunogenicity of different PCV schedules or formulations [17,18] and impact on pneumococcal [19] and NTHi carriage [17], but impacts on hearing have not been included.

Unexpectedly, this report focussing on hearing outcomes found that, contrary to our hypotheses, the children assessed in the +P group had better hearing compared to the +S group at age 18 months. The −3 dB difference in mean hearing level is of unknown clinical value. However, the −19% difference in prevalence of moderate hearing loss and gain of 19% having no hearing loss is likely to benefit many First Nations children, particularly if this is sustained to age 36 months. In addition, around two-thirds of the children assessed at age 18 months met Hearing Australia criteria for hearing assistance [12], and the proportion was again lower in the +P group at age 18 months (62% versus 76%). At later ages, vaccine group differences in hearing persisted. Analysis stratified by the primary course head-to-head and combination schedules (the PREVIX_COMBO RCT) [5] suggested that slightly better hearing in the +P group was present across all primary schedules and was greatest in the SSSP+P versus SSSP+S comparison. The 19% higher prevalence of no hearing loss in the +P group (36%) compared to the +S group (16%) at age 18 months is also noteworthy as our surveillance over many years has consistently found less than 10% children are without OM at this age [3,20]. These unique findings are difficult to explain and should be interpreted with caution as not all randomised participants received hearing assessments.

A major limitation of our study is small sample size for this secondary outcome [10]. As mentioned, the low numbers receiving hearing tests was almost solely related to delays in implementation of hearing tests, rather than selection of participants based on certain characteristics or preferences. We show that baseline characteristics and risk factors of participants receiving hearing tests were not different between vaccine groups at any age. The high event rate somewhat compensates for low numbers and may increase confidence in the apparent vaccine group difference in hearing detected at primary endpoint age 18 months. Given the paucity of published data from vaccine trials to support or refute our findings, further investigation of biological plausibility is warranted, particularly as higher valency vaccines become available [21].

Our systematic review identified an almost complete absence of published contemporary (PCVera) data on the prevalence and persistence of hearing loss in very young First Nations children [22]. In addition, data from the NT hearing outreach services show that only 11% of services (either audiology, Clinical Nurse Specialist, or ear, nose, and throat (ENT) teleotology) were provided to children under age 3 years [23]. Of 1,741 First Nations children who received audiology services, 56 (3%) were under age 3 years and of these 17 (31%) had some level of hearing loss.

The systematic review [22] and national OM guidelines [14] also highlight the paucity of prevalence studies and absence of published intervention studies that address this crisis. In 2022 and 2023, the first community-based prevalence studies of hearing loss in First Nations children living in urban settings were published; one identified that at age 12 months, 69% of 67 children tested had hearing loss, including 24 (36%) with moderate hearing loss [24]. The second, larger study, which excluded children under age 3 years, obtained hearing data from 1,087 (median age 8·2 years) of 1,669 children enrolled; 279 (25·7%) had hearing loss [25]. Neither study was an evaluation of hearing loss rehabilitation or prevention strategies.

Using combined vaccine group data, our key findings identified for the first time the high prevalence of persistent and fluctuating mild to moderate hearing loss throughout early childhood, from 75% at age 12 months to 53% at age 36 months. In particular, the high prevalence of moderate hearing loss, defined by the World Health Organization as disabling for children [11], which was 42% at age 12 months, declining slowly to 19% at age 36 months.

These findings place many children in need of hearing assistance according to the Hearing Australia threshold of ≥23·3 dB [12]. Also, according to national OM guidelines [14], at ages 12 to 30 months, around 30% children met criteria for ENT consultation, based on the mean hearing level in the better ear of >30 dB.

Transitions in level of hearing loss between consecutive 6-monthly visits, despite stable prevalence rates at each time point, confirm the fluctuating nature of conductive hearing loss in this age group. This has practical implications that support the shift from screening programs to surveillance, commencing within first year of life [26].

A strength of our study is that all hearing tests were performed in sound-treated facilities by paediatric audiologists at scheduled study visits, regardless of clinical presentation. However, the majority of hearing tests were VROA for which the minimum test level varies according to test environment and child. If the minimum test level available was 20 to 25 dB, children with a true hearing level below test limits (i.e., no hearing loss) could be misidentified as having mild hearing loss using our study definitions, producing a false positive and overinflating the prevalence of mild hearing loss as determined by VROA. The prevalence of moderate (disabling) hearing loss is likely less susceptible to overinflation, and, at over 30% at every age until age 36 months, remains plausible and unacceptable.

From primary healthcare to specialist services, there are huge shortfalls that disrupt a child’s hearing care pathway [27]. At December 2022, there were 3,265 Indigenous children and young people on the wait list for fly-in/fly-out audiology outreach services and 2,284 on the ENT teleotology wait list [23]. As mentioned, in 2022, very few outreach services were for children under age 3 years, and ENT surgeries were rarely performed (4% of all ENT services were for grommets) [23].

This report of the extraordinarily high prevalence of preventable hearing loss in First Nations children highlights the need for effective social and public health reforms with greater investment in interventions that target social determinants, namely, more preventative models [28]. Others have demonstrated the inequalities in ENT service provision between Aboriginal and non-Aboriginal children, attributed to socioeconomic status and geographical isolation [29]. In the United States, social deprivation limits children’s access to appropriate medical and surgical treatment for their ear disease and increases odds of severe complications [30]. While ear and hearing problems are underserviced, effective prevention strategies are lacking and there is a dearth of efficacy studies that comprehensively investigate the benefits and harms of specialist ENT or audiological interventions for children living in remote regions [31]. Unfortunately studies of interventions that address social determinants will be difficult and few that report OM outcomes have been published or show a benefit [32]. Our research program is currently evaluating a primary healthcare workforce enhancement strategy (the Hearing for Learning Initiative) of remote on-country training and employment of ear health facilitators, which could provide a sustainable, culturally appropriate, and safe model to detect and prevent disabling hearing loss and support children who have hearing problems [33,34].

In conclusion, our discovery of a previously unrealised potential benefit of pneumococcal conjugate vaccination in reducing hearing loss should be investigated further as new expanded valency pneumococcal vaccines are developed. We strongly recommend more rigorous innovative trial designs, incorporating broader outcomes such as hearing loss and developmental milestones. This will improve understanding of the full impact of vaccination on all outcomes of importance, particularly those with downstream socioeconomic benefits for disadvantaged populations.

## Ethics approval and consent to participate

Ethical approval has been obtained from Human Research Ethics Committees of the Northern Territory Department of Health and Menzies School of Health Research (NHMRC Reg no: EC00153), the Central Australian HREC (NHMRC Reg no: EC00155), and West Australian Aboriginal Health Ethics Committee (WAAHEC-377-12/2011). Parents or guardians provided signed informed consent for their infant’s participation.

## Data safety monitoring

The study was overseen by an independent Data Safety and Monitoring Board (iDSMB).

## Supporting information

S1 CONSORT Checklist Citation: Schulz KF, Altman DG, Moher D, for the CONSORT Group. CONSORT 2010 Statement: updated guidelines for reporting parallel group randomised trials. BMC Medicine. 2010;8:18. 2010 Schulz et al. This is an Open Access article distributed under the terms of the Creative Commons Attribution License (http://creativecommons.org/licenses/by/2.0), which permits unrestricted use, distribution, and reproduction in any medium, provided the original work is properly cited. *We strongly recommend reading this statement in conjunction with the CONSORT 2010 Explanation and Elaboration for important clarifications on all the items. If relevant, we also recommend reading CONSORT extensions for cluster randomised trials, noninferiority and equivalence trials, nonpharmacological treatments, herbal interventions, and pragmatic trials. Additional extensions are forthcoming: for those and for up-to-date references relevant to this checklist, see www.consort-statement.org.
(DOC)

## Abbreviations

CI: confidence interval
ENT: ear, nose, and throat
NT: Northern Territory
NTHi: nontypeable *Haemophilus influenzae*
OM: otitis media
PCV: pneumococcal conjugate vaccine
PCV13: 13-valent pneumococcal conjugate vaccine
PHiD-CV10: 10-valent pneumococcal *Haemophilus influenzae* protein D conjugate vaccine
RCT: randomised controlled trial
VROA: Visual Reinforcement Observation Audiometry
4fa: 4-frequency average
